# Signals That Tell Fly Neurons to Extend or Retract

**DOI:** 10.1371/journal.pbio.0040391

**Published:** 2006-10-10

**Authors:** Françoise Chanut

During metamorphosis, fruit fly larvae shed the maggot sheath that had confined their life to a tedious creepy-crawl and don legs, wings, and a pair of big, buggy eyes to explore the third dimension and broaden their horizons. A less conspicuous, but just as wondrous transformation also takes place in their brains. There, new neurons are born and new neural networks are established, which the insects will use to make sense of the environment sampled by their shiny new limbs. How neurons select the appropriate partners among myriad candidates is a long-standing question in neurobiology. And what powers the growth of their axons, the long extensions with which they probe the brain until they reach the right target, is also mostly unknown. Mohammed Srahna, Bassem Hassan, and their colleagues have tackled these issues by genetically manipulating a small cluster of neurons whose axons carve their way through the optic lobe of the fly brain during metamorphosis.

**Figure:**
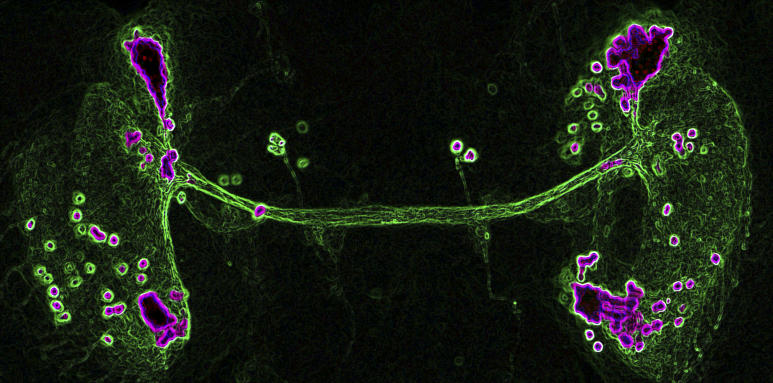
The dorsal cluster neurons (magenta) and their neurites (green) forming a commissure (a bridgeway) across the adult fly brain and branching bilaterally over the fly visual centers. (Image: Lee Fradkin, Jasprien Noordermeer, and Bassem Hassan)

The neurons, known as dorsal cluster neurons (DCNs), lie in clusters of approximately 40 near the top of each brain hemisphere. At the end of larval life, they start extending a long axonal branch across the brain, all the way to the opposite optic lobe (contralateral branch). Using a gene construct specifically expressed in the DCN *(ato-Gal4-14a)*, the researchers marked the neurons with a fluorescent protein to follow their axons’ trajectories. A third of the way through metamorphosis, most contralateral branches (30–40) had passed the lobula and reached the medulla, two neighboring brain areas that integrate neuronal inputs coming from the retina. By the adult stage, however, only a dozen contralateral branches remain connected to the medulla. The researchers suspected that the other branches degenerated in the late phases of metamorphosis. Indeed, they observed shortened DCN axons between lobula and medulla in flies mid-way through metamorphosis. But they found no evidence of axon breakdown, such as swelling or fragmentation. They concluded that most DCN axons retracted back to the lobula after reaching the medulla.

To probe the mechanics of DCN axon growth and retraction, the researchers blocked specific signaling pathways in the DCN by driving the expression of various gene constructs with *ato-Gal4-14a*. Blocking the intracellular signaling protein JNK or a receptor (Fz2) for the extracellular signal Wnt prevented the extension of DCN axons beyond the lobula. Blocking another intracellular signaling protein called Rac1 or the receptor for the extracellular signal FGF caused more DCN axons than normal to innervate the medulla. By simultaneously manipulating several pathways, the researchers determined how the pathways interact to regulate DCN axon growth. For instance, blocking JNK suppressed the excessive medulla innervation caused by blocking Rac1. This observation suggests that JNK is necessary for DCN axons to reach the medulla and that Rac1 normally impairs the activity of JNK.

Based on additional genetic and biochemical analyses, the researchers propose that JNK signaling is the engine that drives the growth of the DCN axons all the way to the medulla. In the medulla, the axons encounter a localized pool of FGF, which causes some of them to retract back to the lobula. FGF signaling acts by facilitating the inhibition of JNK by Rac1 within the DCN. In contrast, Wnt signaling boosts the activity of JNK by blunting the inhibitory effect of Rac1. The researchers propose that Wnt signaling in DCNs allows axons to maintain their connection to the medulla. Extension or retraction of DCN axons might therefore reflect a different balance of the antagonistic effects of FGF/Rac1 and Wnt/JNK signaling. Single-cell mutations suggest that the integration of these signals is happening in each of the 40 DCNs independently, meaning that the global pattern arises as a result of the autonomous action of individual cells. What remains now to be understood is what makes the 12 or so DCNs that stay connected to the medulla strike that balance differently from their 28 counterparts.

